# Diagnostic accuracy of ^99m^Tc-HYNIC-TOC SPECT/CT for detecting osteomalacia-associated tumors

**DOI:** 10.3389/fonc.2023.1228575

**Published:** 2023-07-24

**Authors:** Bo Li, Lili Duan, Xiali Li, Jingqi Shi, Huiqiang Li, Huimin Liu, Xiaoliang Cheng, Xinyu Wu, Yongju Gao

**Affiliations:** ^1^Department of Nuclear Medicine, Henan Key Laboratory of Novel Molecular Probes and Clinical Translation in Nuclear Medicine, Henan Provincial People’s Hospital; Zhengzhou University People’s Hospital, Henan University People’s Hospital, Zhengzhou, China; ^2^Department of Oncology, Henan Provincial People’s Hospital, Zhengzhou University People’s Hospital, Henan University People’s Hospital, Zhengzhou, China; ^3^Department of Pharmacy, The First Affiliated Hospital of Xi’an Jiaotong University, Xi’an, China

**Keywords:** ^99m^Tc-HYNIC-TOC, SPECT/CT, somatostatin receptor, tumor-induced osteomalacia, diagnostic accuracy

## Abstract

**Objectives:**

Tumor-induced osteomalacia (TIO) is a rare acquired paraneoplastic disorder characterized by hypophosphatemia resulting from tumor-secreted fibroblast growth factor-23 (FGF23). Surgical resection of the culprit TIO is the first choice of treatment. However, TIO is difficult to detect with conventional diagnostic tools due to its small size and variable location in the body. Somatostatin receptor scintigraphy (SSR) has recently emerged as a functional molecular imaging choice for TIO detection and localization. This research was undertaken to evaluate the efficacy of ^99m^Tc-labeled hydrazinonicotinyl-Tyr3-octreotide (^99m^Tc-HYNIC-TOC) SPECT/CT in detecting TIO.

**Methods:**

^99m^Tc-HYNIC-TOC SPECT/CT and the available clinical data of 25 patients with suspected TIO were analyzed retrospectively. The ^99m^Tc-HYNIC-TOC SPECT/CT findings were compared with the post-surgical pathology diagnosis and clinical follow-up results.

**Results:**

Using ^99m^Tc-HYNIC-TOC SPECT/CT, suspicious tumors were found in 18 of the 25 patients, and 15 of them underwent surgical resection. The post-operative pathology confirmed a TIO in those 13 patients whose symptoms and biochemical anomalies gradually resolved after the surgery. The remaining five patients were finally considered false positives. Moreover, the ^99m^Tc-HYNIC-TOC SPECT/CT results were negative in seven patients, with six patients being true negative (4 patients were diagnosed with acquired Fanconi syndrome and 2 patients responded well to conservative therapy) and one being false negative. Therefore, the sensitivity and specificity values of ^99m^Tc-HYNIC-TOC SPECT/CT in the evaluation of TIO were 92.9% (13/14) and 54.5% (6/11), respectively. The overall accuracy of ^99m^Tc-HYNIC-TOC SPECT/CT for detecting TIO was 76.0% (19/25).

**Conclusions:**

The ^99m^Tc-HYNIC-TOC SPECT/CT is an accurate imaging modality for locating culprit tumors in TIO.

## Introduction

Tumor-induced osteomalacia (TIO), also known as oncogenic osteomalacia, is a rare paraneoplastic syndrome mainly caused by small phosphaturic mesenchymal tumors (PMTs) that secrete fibroblast growth factor-23 (FGF-23) (1). FGF-23 is a phosphatonin that regulates renal phosphate handling and vitamin D homeostasis. Thus, a high level of FGF23 causes renal phosphate wasting, hypophosphatemia, and decreased serum active vitamin D. Chronic hypophosphatemia ultimately results in osteomalacia (1, 2). Typically, patients with TIO present with clinical findings of progressive muscle weakness, bone pain, and recurrent fractures. The primary treatment option is surgical resection of the culprit tumor. However, localization of the causative tumor is challenging due to its small size and rare location. In addition, because of the slow-growing nature of TIO tumors, local symptoms are frequently overshadowed by the severe systemic consequences of osteomalacia (1, 2). This often delays the correct diagnosis and localization of tumors. Consequently, locating the culprit tumor remains the most crucial aspect of treating TIO.

With relatively low sensitivity and specificity, anatomy-based imaging modalities, such as X-rays, computed tomography (CT), and magnetic resonance imaging (MRI), have been used for the localization of these rare tumors (1, 2). Recently, functional molecular imaging with a somatostatin receptor (SSTR)-based strategy has emerged as a sensitive diagnostic method for TIO localization (1–5). The SSTR-targeting agent, octreotide, was labeled with PET tracer Ga-68, which has gained popularity for TIO localization due to its better-quality images in recent years (3–11). The use of SPECT-based tracers, such as ^99m^Tc-HYNIC-TOC, for the detection of TIO was also demonstrated. A few studies have reported promising results when using the ^99m^Tc-HYNIC-TOC whole-body scan to detect TIO (12, 13).

In the present study, the diagnostic performance of ^99m^Tc-HYNIC-TOC whole-body scan with SPECT/CT in detecting TIO was retrospectively analyzed.

## Materials and methods

### Patients

From June 2017 to March 2022, the medical records of 25 patients (14 men and 11 women) with suspected TIO were retrospectively analyzed. The inclusion criteria were clinical symptoms (fatigue, bone pain, and/or pathological fractures) and biochemical anomaly (low serum phosphate) compatible with TIO. Each patient was evaluated using ^99m^Tc-HYNIC-TOC SPECT/CT. The ^99m^Tc-HYNIC-TOC scintigraphy findings were compared with the other imaging methods (CT, MRI), the results of post-surgical pathology diagnosis, and clinical follow-up. Based on the principles of the Declaration of Helsinki, the ethics committee of the Henan Provincial People’s Hospital & Zhengzhou University People’s Hospital approved this study (Approval number: 109-10-15). All patients gave written permission to use their data and informed consent for anonymous publication.

### ^99m^Tc-HYNIC-TOC scintigraphy

^99m^Tc-HYNIC-TOC was synthesized and labeled according to published methods (12, 14). Whole-body imaging was acquired at one and four hours after intravenous injection of 555-740 MBq (15-20 mCi) of ^99m^Tc-HYNIC-TOC via a dual-head SPECT/CT camera (D670, GE Healthcare). Whole body planar scintigraphy (anterior and posterior) was acquired using a low-energy high-resolution (LEHR) collimator with a matrix of 256 × 1024 and a scan speed of 10 cm/min. SPECT/CT on the thorax, abdomen, and suspected positive regions was performed for each enrolled patient at four hours post-radiopharmaceutical administration. Imaging parameters for SPECT were: 6 degrees angular resolution and 30s per step with a 256 × 256 matrix. Low-dose CT scan parameters were as follows: 130 kV and 25 references mAs modulation. CT data were reconstructed at 5 mm slice thicknesses using smooth (B41s) kernels. SPECT, CT, and fused imaging of ^99m^Tc-HYNIC-TOC scan were analyzed using MedEx software (MedEx Medical Ltd, Beijing, China). Masked reading was performed by two senior and one junior nuclear medicine physicians who were unaware of clinical information. The activity of foci higher than adjacent normal tissues and not associated with physiological uptake was regarded as a positive finding.

## Results

### Patients’ characteristics and final diagnoses

Twenty-five patients (14 men and 11 women) with suspected TIO were retrospectively analyzed. The clinical characteristics, ^99m^Tc-HYNIC-TOC imaging results, as well as histopathological findings are summarized in **Table 1**. The patients’ ages ranged from 29 to 77 years (median 53 years). All patients presented with clinical symptoms associated with fatigue, bone pain, tenderness, and biochemical abnormalities suspicious of TIO. As depicted in **Table 1**, the mean serum phosphate concentrations were 0.52 mmol/L (the normal reference range was 0.85–1.51 mmol/L). The serum alkaline phosphatase (ALP) level was higher in 17 patients. Bone densitometry revealed osteopenia or osteoporosis in 12 patients. From the onset of symptoms to the completion of the ^99m^Tc-HYNIC-TOC SPECT/CT, the average time for all patients was 35 months (range 9-108 months).

**Table 1:** The patients’ characteristics, ^99m^Tc-HYNIC-TOC imaging results, and histopathological findings of 25 patients.

Case	Age/ gender	Symptoms	Symptom duration (months)	P (mmol/L)	ALP (U/L)	Ca (mmol/L)	^99m^Tc-HYNIC-TOC uptake site	Histology	Results of ^99m^Tc-HYNIC-TOC
1	47/F	Lower limbs pain and muscle weakness	15	0.42	122	2.15	Left heel	PMT	TP
2	41/M	Muscle weakness and inability to walk	60	0.51	438	2.30	Right foot	PMT	TP
3	52/F	Diffuse body pain	15	0.56	171	2.32	Not detected	–	TN
4	67/F	Left foot pain	24	0.79	152	2.15	Left calcaneus	Neuroendocrine tumor	FP
5	47/F	Right hip pain and muscle weakness	30	0.39	472	2.30	Right femoral head	PMT	TP
6	56/M	Left hip pain	24	0.72	152	2.14	Not detected	–	TN
7	53/F	Muscle weakness	60	0.45	178	2.31	Right forearm	PMT	FN
8	60/M	Diffuse body pain	36	0.42	230	2.10	Right knee	PMT	TP
9	58/M	Hips and lower limbs pain	54	0.39	558	2.16	Left thigh muscle gap	PMT	TP
10	49/M	Back and lower limbs pain	18	0.48	53	2.47	Not detected	–	TN
11	53/M	Lower limbs pain and poor walking	24	0.61	237	2.42	Lower end of the left femur	PMT	TP
12	54/M	Diffuse body pain	18	0.55	116	2.35	Left maxillary sinus	PMT	TP
13	67/M	Muscle weakness	48	0.42	73	2.22	Right gluteal	–	FP
14	50/M	Diffuse bone pain	36	0.38	210	2.22	Right buttock muscle gap	Hemangioma	TP
15	42/F	Chest and back pain	24	0.53	106	2.13	Right mandible	PMT	TP
16	54/F	Lower back pain and muscle weakness	40	0.45	157	2.30	Right lower back	Necrotic tissue	FP
17	72/M	Diffuse body pain and muscle weakness	108	0.65	325	2.23	Fourth lumbar vertebra	PMT	TP
18	52/M	Muscle weakness	30	0.62	253	2.19	Not detected	–	TN
19	29/F	Back pain and muscle weakness	9	0.57	175	2.38	Eleventh thoracic vertebra	–	FP
20	77/F	Diffuse body pain and muscle weakness	18	0.51	95	2.07	Not detected	–	TN
21	51/F	Diffuse bone pain and muscle weakness	13	0.29	172	2.15	Right thigh	Giant cell tumor	TP
22	49/M	Muscle weakness	30	0.65	92	2.45	Clivus	–	FP
23	61/F	Diffuse bone pain	72	0.53	322	1.95	Right mandible	PMT	TP
24	42/M	Diffuse body pain	12	0.73	82	2.51	Not detected	–	TN
25	52/M	Diffuse bone pain and muscle weakness	66	0.38	445	2.26	Right upper back	PMT	TP

Normal reference range: Phosphorus (P) 0.85-1.51 mmol/L; alkaline phosphatase (ALP) 45-125 U/L; Calcium (Ca) 2.11-2.52 mmol/L.

M, male; F, female; PMT, phosphaturic mesenchymal tumor; TP, true positive; FP, false positive; TN, true negative; FN, false negative.

**Figure 1:**
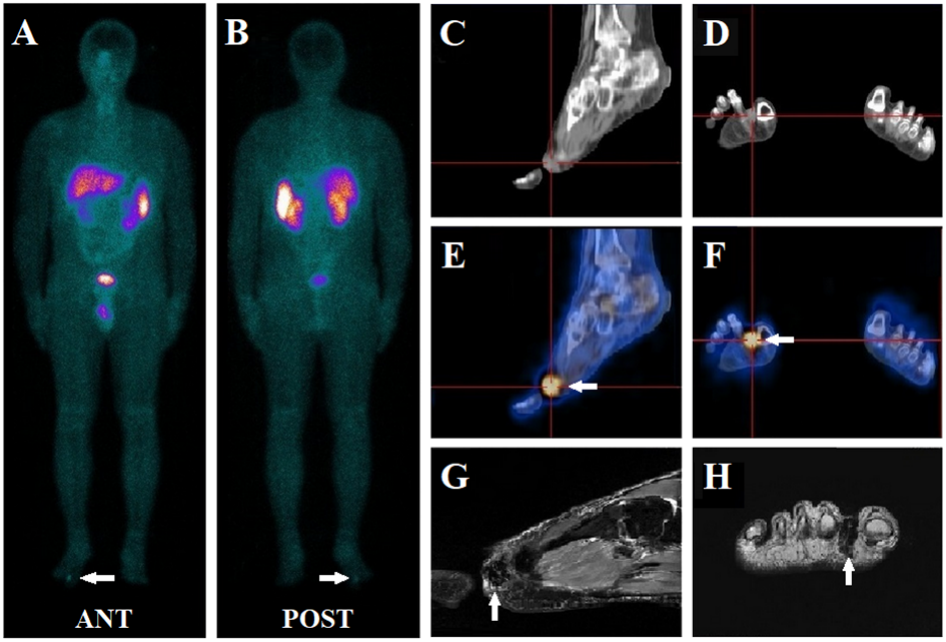
A 41-year-old male presented with muscle weakness, inability to walk, and hypophosphatemia for more than five years. A whole-body ^99m^Tc-HYNIC-TOC scan **(A, B)** was conducted to evaluate possible TIO, which revealed abnormal activity in the right foot (white arrow). Further, SPECT/CT images demonstrated an intense focal uptake (white arrow) between toes 1 and 2 in the right feet **(E, F)**, which was visualized as a soft tissue density nodule on the CT scan **(C, D)**. Subsequent MRI (T2-weighted image) depicted a small mass (white arrow) in the region seen on ^99m^Tc-HYNIC-TOC SPECT/CT **(G, H)**. A further physical examination of the subcutaneous area confirmed the presence of a nodule. The patient underwent surgery, and the histology indicated a benign phosphaturic mesenchymal tumor without malignancy. His symptoms resolved, and his serum phosphate levels increased from 0.51 mmol/L pre-surgically to 0.96 mmol/L post-operatively.

**Figure 2:**
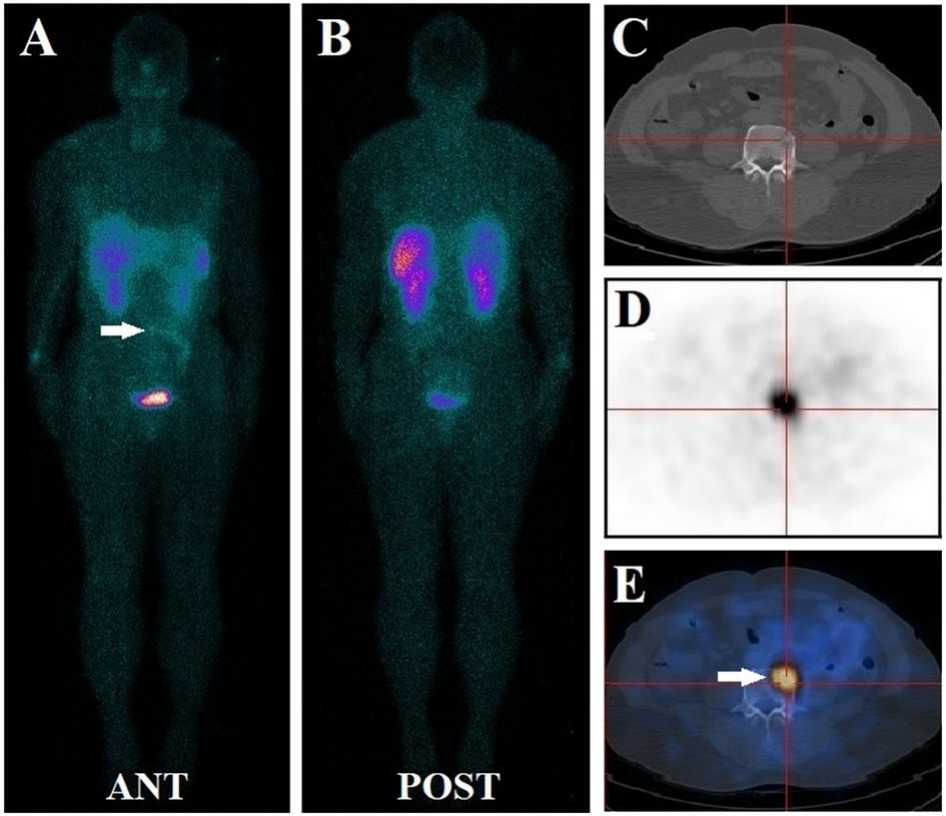
A 72-year-old male had diffuse bone pain and muscle weakness for nine years. Before this study, his serum phosphate level was lower than normal at 0.65 mmol/L. the patient underwent a ^99m^Tc-HYNIC-TOC whole-body planar scan, and no evident focus of elevated activity was identified **(A, B)**. Subsequent thoracic and abdominal SPECT/CT images revealed small ^99m^Tc-HYNIC-TOC positive foci in the fourth lumbar vertebra (white arrow) **(C–E)**. The foci (white arrow) were missed during planar scanning due to radio-retention interference from adjacent bowels. The diagnosis of phosphaturic mesenchymal tumor was confirmed after the lesion was surgically removed. The long-term symptoms of this patient disappeared promptly, and his post-surgical serum level increased to normal at 1.21 mmol/L.

**Figure 3:**
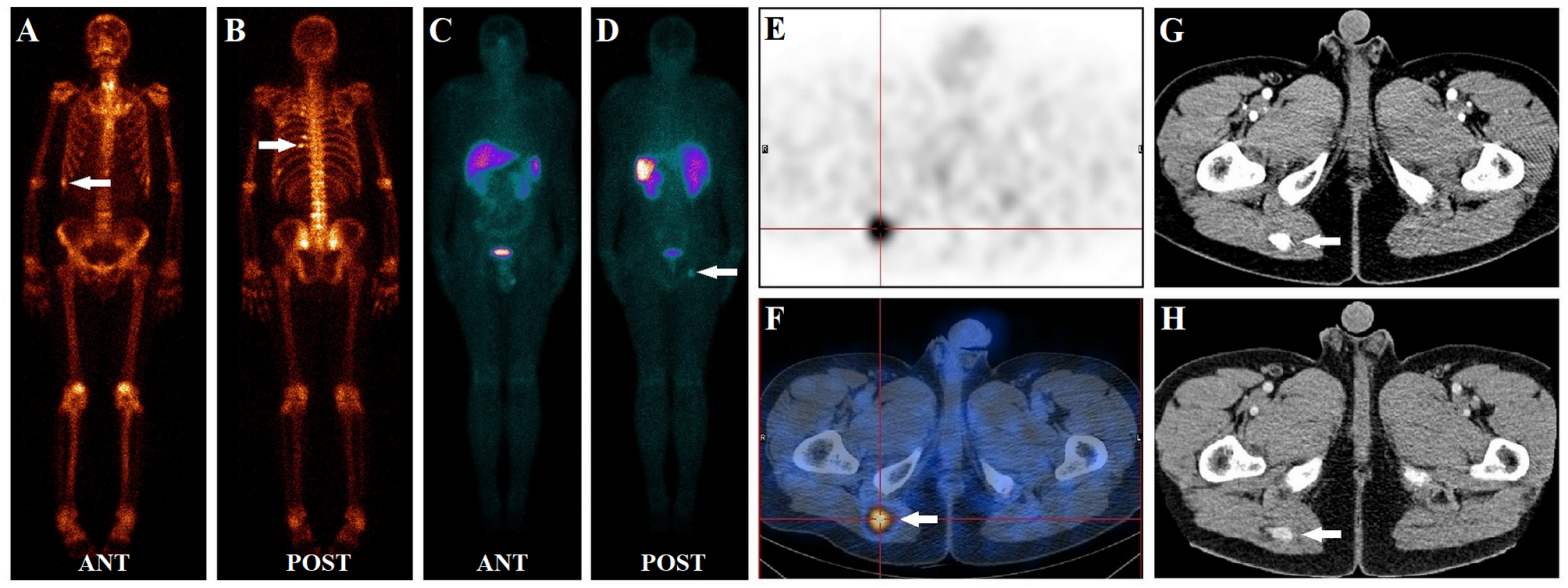
A 50-year-old male had diffuse bone pain for three years. Laboratory tests revealed severe hypophosphatemia (serum phosphate level of 0.38 mmol/L, normal reference range of 0.85–1.51 mmol/L). During a ^99m^Tc-methylene diphosphonate bone scan **(A, B)**, multiple foci of intense MDP activity were identified in the skeleton (white arrow), suggesting fractures. In the ^99m^Tc-HYNIC-TOC whole-body planar scan **(C, D)**, abnormal activity foci were observed in the right buttock (white arrow). Subsequent SPECT/CT images revealed focal uptake (white arrow) in the right buttock muscle gap **(E, F)**. Contrast-enhanced CT depicts that the tissue nodule lesion (white arrow) was strongly enhanced **(G, H)**. Interestingly, none of the fracture sites found on a bone scan had elevated ^99m^Tc-HYNIC-TOC activity. After excision of the suspicious lesion, the histology was hemangioma without malignant features. Post-surgically, the patient ’s symptoms gradually improved, and his serum phosphate level returned to normal at 1.03 mmol/L.

**Figure 4:**
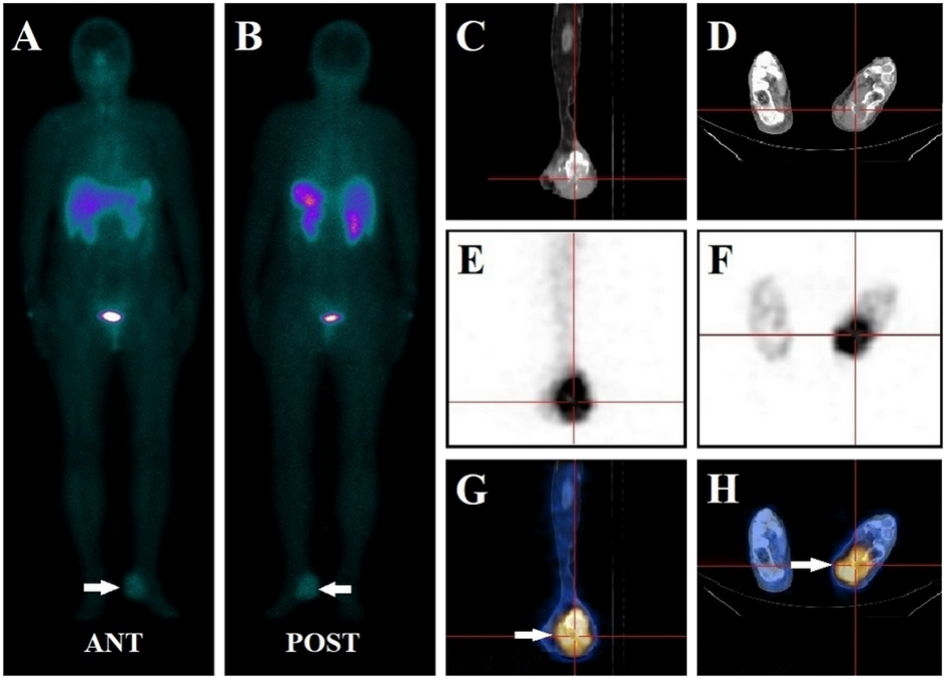
A 67-year-old female had left foot pain for more than two years. Her serum phosphate level was 0.79 mmol/L at presentation. The whole-body ^99m^Tc-HYNIC-TOC scan **(A, B)** demonstrated abnormal activity foci in the left foot (white arrow). SPECT/CT images revealed focal intense uptake in the left calcaneus bone in SPECT **(E, F)** and fused SPECT-CT images (white arrow) **(G, H)**. CT scans **(C, D)** identified bone destruction with associated soft tissue swelling of the left calcaneus. The lesion was completely excised and confirmed to be a well-differentiated G2 neuroendocrine tumor. Pain symptoms ceased, and the patient’s serum phosphate levels increased to 1.17 mmol/L after oral phosphate supplementation. Thus, ^99m^Tc-HYNIC-TOC SPECT/CT detected foci were considered a false positive finding.

In 18 patients, suspicious tumors were detected using ^99m^Tc-HYNIC-TOC SPECT/CT imaging. Surgical resection was performed on 15 patients. In 11 cases, the post-surgical pathology confirmed phosphaturic mesenchymal tumors (PMTs), including mixed connective tissue variants (**Figures 1**, **2**), while the other two patients had giant cell tumor and hemangioma (**Figure 3**), respectively. After surgery, these patients’ symptoms gradually improved over several months. Due to the predominance of necrotic tissue in the surgical specimen of one patient, the tumor type could not be determined. In the other one operated patient, the post-operative pathological diagnosis was a well-differentiated G2 neuroendocrine tumor, as illustrated in **Figure 4**. After surgery, the patient’s pain symptoms subsided, and their serum phosphate levels returned to normal after oral phosphate supplementation. Therefore, ^99m^Tc-HYNIC-TOC detected foci were considered false positive findings.

In three non-operated patients, ^99m^TC-HYNIC-TOC detected TIO-suspicious foci, but the clinical presentation ultimately refuted this diagnosis. The symptoms of the three patients were promptly relieved after oral phosphate and calcitriol supplementation. During the 12 months of follow-up, none of these three patients had hypophosphatemia, excluding a TIO diagnosis.

Among the seven patients with negative ^99m^Tc-HYNIC-TOC scans, six were finally diagnosed with other causes of hypophosphatemia (acquired Fanconi syndromes, n = 4) or responded well to conservative therapy (phosphate and calcitriol supplementation, n = 2). Physical examination revealed a subcutaneous nodule on a patient’s right forearm, which was later determined to be PMT after surgical removal. Accordingly, the result of this patient was considered false negative.

### The accuracy of the ^99m^Tc-HYNIC-TOC SPECT/CT imaging

^99m^Tc-HYNIC-TOC SPECT/CT detected the culprit tumors in 18 of the 25 patients. Thirteen of these 18 patients were confirmed pathologically as true positives, while the remaining five were found to be false positives. Among the seven patients with negative ^99m^Tc-HYNIC-TOC images, six were true negatives, and one was a false negative. The sensitivity and specificity values of ^99m^Tc-HYNIC-TOC SPECT/CT in the evaluation of TIO were, therefore, 92.9% (13/14) and 54.5% (6/11), respectively. The overall accuracy was 76.0% (19/25).

### Locations of the culprit tumors

**Figure 5:**
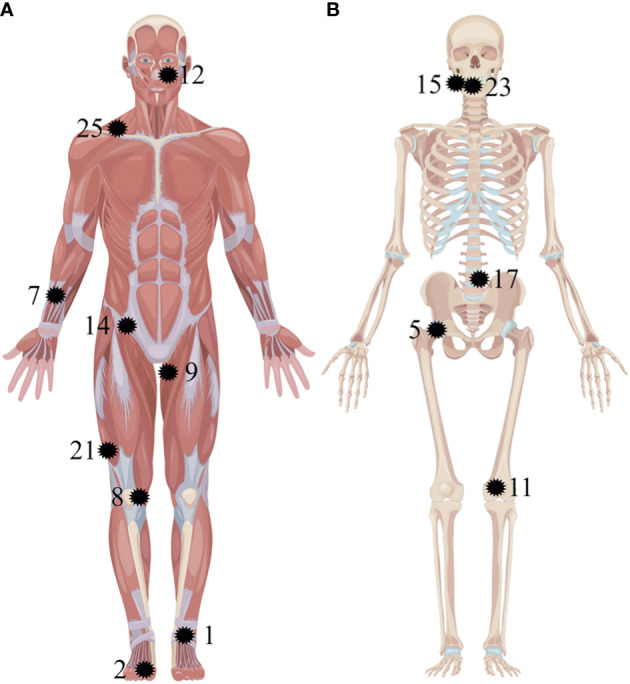
Anatomic localization of pathologically confirmed culprit tumors in 14 patients. The tumors are located in the soft tissues **(A)** and the bones **(B)** (as pointed out by the black shape). The numbers correspond to the patients listed in **Table 1**.

As summarized in **Figure 5**, the culprit tumors could be either in the soft tissues (**Figures 1**, **3**) or the bones (**Figure 2**). The most common tumor locations were within the soft tissues (9/14), which were distributed in the craniofacial region (one case), torso (two cases, **Figure 3**), upper (one case), and lower extremities (five cases, **Figure 1**). The remaining five cases of bone tumors were found in the right mandible (two cases), the fourth lumbar vertebra (one case, **Figure 2**), the right femoral head (one case), and the lower end of the left femur (one case).

## Discussion

The diagnosis of TIO remains challenging when serum phosphate levels are chronically low in the setting of progressive muscle weakness, bone pain, and recurrent fractures (1, 2). Due to their small size and random distribution, tumors can be challenging to locate and pinpoint. Conventional diagnostic approaches, such as ultrasound, CT, MRI, and clinical examination, are insufficient for tumor detection (1, 2).

Recent studies have highlighted the role of functional imaging in the early diagnostic workup of TIO. Multiple mesenchymal tissue-derived tumors, such as PMT, are known for overexpression of SSTR, especially the SSTR2 receptor. Thus, SSTR scintigraphy has been identified as an effective diagnostic method for localizing causative tumors (3–13). In the present study, the utility of a ^99m^Tc-HYNIC-TOC whole-body scan with SPECT/CT in detecting culprit tumors for TIO was retrospectively analyzed. Our results illustrated that ^99m^Tc-HYNIC-TOC SPECT/CT has a high diagnostic performance for localizing causative tumors, with a sensitivity of 92.9% (13/14), a specificity of 54.5% (6/11), and an accuracy of 76.0% (19/25).

Despite the limited number of studies, a few have previously demonstrated that ^99m^Tc-HYNIC-TOC scintigraphy has a valuable diagnostic yield on TIO, with high sensitivity, specificity, and accuracy (12, 13, 15). Zuo et al. (13) reported a comparable detection rate (81.8%, 9/11). In the largest ^99m^Tc-HYNIC-TOC image study, Jing et al. (12) demonstrated an accuracy of 93.4% (171/183) for detecting culprit tumors in 183 patients, which was higher than the accuracy (76.0%) from our investigation. This discrepancy might be attributed to the difference in the number of subjects enrolled. A similarly high detection rate was also observed in another smaller study by Jadhav et al. (100%, 6/6) (15).

For decades, ^111^ in-labeled octreotides SPECT/CT imaging has been available on the market for the detection of culprit tumors in the clinical setting (2, 3, 16). One of the pioneering studies (2) showed a sensitivity of 71.4% (5/7) in seven patients who suspected TIO, which was lower than the sensitivity (92.9%) of our findings. A significantly lower detection rate of 36.3% (4/11) was observed in the comparative study by El-Maouche et al. (3). The reasons for this discrepancy might be partly due to differences in the tumor uptake of imaging agents. Decristoforo et al. demonstrated that ^99m^Tc-HYNIC-TOC has higher retained somatostatin receptor binding affinities than ^111^In-octreotide (17).

Recently, Ga-68 labeled somatostatin analogs (such as ^68^Ga-DOTA-TATE, ^68^Ga-DOTA-NOC, and ^68^Ga-DOTA-TOC) PET/CT imaging have demonstrated a favorable diagnostic performance for localizing offending tumors in TIO (3, 11). ^68^Ga-DOTA-TATE are the most frequently reported in current clinical practice. As displayed in **Table 2**, several studies have reported high TIO detection rates with ^68^Ga-DOTA-TATE PET/CT, ranging from 83% to 100% (6, 7, 15, 20, 23–27). Zhang et al. (8) and Yu et al. (22) reported similar high sensitivity, specificity, and accuracy in the detection of TIO. In contrast, several studies demonstrated a similar high diagnostic accuracy of ^68^Ga-DOTA-NOC and ^68^Ga-DOTA-TOC for the localization of TIO (4, 5, 11, 28, 29). Nevertheless, three series have reported less promising detection rates of 55%, 53%, and 57%, respectively, with ^68^Ga-DOTA-TATE, ^68^Ga-DOTA-NOC, and ^68^Ga-DOTA-TOC PET/CT (3, 9, 10).

**Table 2:** Summary of series (N > 2) reporting nuclear medicine imaging for detecting tumor-inducing osteomalacia.

Imaging modality	N	Study Design	Diagnostic performance	Reference
^111^In-pentetreotide	7	Prospective	Detection rate = 5/7 = 71.4%	Jan de Beur et al.(2002) (2)
	31	NR	Se = 18/19 = 95%, Sp = 7/11 = 64%, Acc = 25/30 = 83%	Chong et al. (2013) (16)
	11	Prospective	Detection rate = 4/11 = 36%	EI-Maouche et al. (2016) (3)
^99m^Tc-HYNIC-TOC	183	Retrospective	Se = 69/80 = 86%, Sp = 102/103 = 99%, Acc = 171/183 = 93%	Jing et al. (2013) (12)
	6	Retrospective	Detection rate = 6/6 = 100%	Jadhav et al. (2014) (15)
	11	Retrospective	Detection rate = 9/11 = 82%	Zuo et al. (2017) (13)
	56	Retrospective	Se = 24/41 = 59%, Sp = 13/15 = 87%, Acc = 37/56 = 66%	Zhang et al. (2020) (18)
	25	Retrospective	Se = 13/14 = 93%, Sp = 6/11 = 55%, Acc = 19/25 = 76%	Present study
^18^F-FDG	5	Retrospective	Detection rate = 4/5 = 80%	Jagtap et al. (2011) (19)
	27	NR	Se = 14/16 = 88%, Sp = 4/11 = 36%, Acc = 18/27 = 67%	Chong et al. (2013) (16)
	8	Retrospective	Detection rate = 4/8 = 50%	Jadhav et al. (2014) (15)
	4	Retrospective	Detection rate = 2/4 = 50%	Agrawal et al. (2015) (20)
	11	Prospective	Detection rate = 4/11 = 36%	EI-Maouche et al. (2016) (3)
	8	Prospective	Detection rate = 7/8 = 88%	Jain et al. (2016) (21)
	13	Retrospective	Se = 6/8 = 75%, Sp =4/5 = 80%, Acc = 10/13 = 77%	Yu et al. (2021) (22)
^68^Ga-DOTA-TATE	6	Retrospective	Detection rate = 6/6 = 100%	Clifton-Bligh et al. (2013) (6)
	7	Retrospective	Detection rate = 7/7 = 100%	Jadhav et al. (2014) (15)
	5	Retrospective	Detection rate = 5/5 = 100%	Breer et al. (2014) (7)
	6	Retrospective	Detection rate = 5/6 = 83%	Agrawal et al. (2015) (20)
	43	Retrospective	Se = 32/32 = 100%, Sp = 10/11 = 91%, Acc = 42/43 = 98%	Zhang et al. (2015) (8)
	11	Prospective	Detection rate = 6/11 = 55%	EI-Maouche et al. (2016) (3)
	8	Prospective	Detection rate = 8/8 = 100%	Satyaraddi et al. (2017) (23)
	37	Retrospective	Detection rate = 37/37 = 100%	Zhang et al. (2018) (24)
	54	Retrospective	Detection rate = 53/54 = 98%	Ding et al. (2018) (25)
	56	Retrospective	Se = 39/41 = 95%, Sp = 9/15 = 60%, Acc = 40/56 = 71%	Zhang et al. (2020) (18)
	4	Retrospective	Detection rate = 4/4 = 100%	Long et al. (2021) (26)
	13	Retrospective	Se = 8/8 = 100%, Sp = 4/5 = 80%, Acc = 12/13 = 92%	Yu et al. (2021) (22)
	19	Prospective	Detection rate = 18/19 = 95%	Hou et al. (2022) (27)
^68^Ga-DOTA-NOC	3	NR	Detection rate = 3/3 = 100%	Ho et al. (2015) (28)
	10	NR	Detection rate = 9/10 = 90%	Bhavani et al. (2016) (4)
	17	Retrospective	Detection rate = 9/17 = 53%	Singh et al. (2017) (9)
	21	Retrospective	Se = 16/17 = 94%, Sp =3/4 = 75%, Acc = 19/21 = 91%	He et al. (2020) (29)
^68^Ga-DOTA-TOC	14	Retrospective	Se = 8/11 = 73%, Sp =2/3 = 63%, Acc = 10/14 = 71%	Paquet et al. (2018) (5)
	35	Retrospective	Detection rate = 20/35 = 57%	Kato et al. (2021) (10)
	12	Retrospective	Se = 7/7 = 100%, Sp = 4/5 = 80%, Acc = 11/12 = 92%	Lee et al. (2021) (11)
^68^Ga-DOTA-JR11	19	Prospective	Detection rate = 11/19 = 58%	Hou et al. (2022) (27)
^18^F-AlF-NOTA-octreotide	17	Retrospective	Se = 14/16 = 88%, Sp = 1/1 = 100%, Acc = 15/17 = 88%	Long et al. (2021) (26)

N, number of patients; NR, not reportd; Se, sensitivity; Sp, specificity; Acc, accuracy.

Recently, a few new positron-labeled octreotide analogs have been attempted to diagnose TIO. Long et al. (26) demonstrated a sensitivity of 87.5%, a specificity of 100%, and an accuracy of 88.2% for localization of TIO in 14 patients using ^18^F-AlF-NOTA-Octreotide PET/CT. In a prospective study (27), ^68^Ga-DOTA-JR11 was developed as an SSTR2-specific antagonist PET tracer for detecting TIO. ^68^Ga-DOTA-JR11 revealed a considerably lower detection rate (57.9%, 11/19) than ^68^Ga-DOTA-TATE (94.7%, 18/19). This result may be due to different binding affinities: ^68^Ga-DOTA-JR11 has a lower SSTR2 affinity than ^68^Ga-DOTA-TATE.

A direct comparison involving the same patient population between ^68^Ga-DOTA-TATE and ^99m^Tc-HYNIC-TOC were reported by Zhang et al. (18). The diagnostic sensitivity and accuracy of ^99m^Tc-HYNIC-TOC SPECT/CT imaging were 58.5% and 66.1%, respectively, which were lower than the sensitivity (95.1%) and accuracy (71.4%) of ^68^Ga-DOTA-TATE PET/CT imaging. In a different study, Jadhav et al. (15) demonstrated somatostatin receptor-based scans, ^99m^Tc-HYNIC-TOC SPECT/CT and ^68^Ga-DOTA-TATE PET/CT performed equally well for localization of TIO. Ding et al. (25) and Xia et al. (30) reported that mild activity at fracture sites does not significantly affect the accuracy of ^68^Ga-DOTA-TATE PET/CT in detecting causative tumors. As illustrated in **Figure 3**, similar results were observed in our study. Although PET/CT imaging provides high-quality images, ^68^Ga generators are not easily and affordably available to many hospitals in our country. In contrast, ^99m^Tc-HYNIC-TOC is relatively cheap, readily available, and can be performed in any nuclear medicine department equipped with SPECT/CT devices. As mentioned above, there is considerable potential for ^99m^Tc-HYNIC-TOC SPECT/CT to be used in TIO for the localization of culprit tumors.

Using some other functional imaging modalities, such as ^18^F-FDG PET/CT, attempts have already been made to localize the offending tumor (3, 15, 16, 19–22). Compared with somatostatin receptor-based imaging modalities, ^18^F-FDG PET/CT exhibited lower diagnostic accuracy (3, 15, 20). The low diagnostic potential of ^18^F-FDG PET/CT can be attributed to the fact that TIO grows slowly, indicating that the glucose metabolism of these tumor cells is only slightly or not at all increased. Interestingly, Wang et al. (31) revealed that a positive presurgical FDG PET/CT suggests an increased likelihood for possible recurrence of TIO after surgical resection.

According to our patient population, most tumors responsible for TIO were located in the soft tissue (64.3%, 9/14), particularly the lower extremities (35.7%, 5/14). This finding is similar to a previous study using a ^99m^Tc-HYNIC-TOC scan (12). TIO-associated tumors were more commonly found in the lower extremities, so they should be thoroughly examined. In our study, the whole-body scan was performed routinely at one and four hours after the ^99m^Tc-HYNIC-TOC injection to prevent missed diagnoses. In addition, to avoid interference with the physiological distribution of the ^99m^Tc-HYNIC-TOC on the corresponding region in the planar scan, SPECT/CT on the thorax, abdomen, and suspected positive regions was performed for each enrolled patient. Jing et al. (12) found a false negative patient who was missed due to interference with radioactive retention of the bladder in a whole-body planar scan. In our study, one patient did not reveal a ^99m^Tc-HYNIC-TOC positive lesion in the whole-body scan. However, a ^99m^Tc-HYNIC-TOC positive lesion was found on the fourth lumbar vertebra during routine thoracic and abdominal SPECT/CT tomography. During planar imaging, this lesion was missed due to radioretention interference from adjacent bowel, as displayed in **Figure 2**. Because SPECT/CT is superior to planar imaging for detecting subsequent lesions, this was done to maximize sensitivity. Using this method, we found that the sensitivity of detecting TIO was higher than that of a previous study (92.9% vs. 86.3%) (12).

In terms of histopathology, these tumors are ubiquitous benign mesenchymal tumors originating from mesenchymal tissue or mixed connective tissues. Our results depicted that all offending tumors were benign, with 12 patients (12/14, 85.7%) diagnosed with PMT, including a mixed connective tissue variant, while the other two causative tumors were giant cell tumor and hemangioma, respectively. Our study is consistent with previous findings showing a predominance of PMT.

TIO is a rare paraneoplastic syndrome, only 895 cases have been reported until April 2020 (32). During nearly five years of follow-up, we found 13 confirmed TIO patients using ^99m^Tc-HYNIC-TOC SPECT/CT. This result undoubtedly increases our confidence in ^99m^Tc-HYNIC-TOC SPECT/CT imaging for localizing culprit tumors. Since ^99m^Tc-HYNIC-TOC is easily synthesized, cost-effective, and can be performed in most nuclear medicine departments, further popularization and application of ^99m^Tc-HYNIC-TOC will help improve the detection rate of TIO.

Our study has a few limitations. First, this was a retrospective study with a small number of subjects due to the rarity of the disease. These patients were referred to our study by different clinicians who had incomplete medical data, particularly for FGF-23. During the time of this study, the FGF-23 test was not yet available at our institution, even though FGF-23 plays a key role in the pathogenesis of TIO. Second, the accuracy of clinical diagnosis among ^99m^Tc-HYNIC-TOC SPECT/CT negative patients remains unclear, and it has not been established whether these patients had TIO or other diseases. Because of these limitations, further studies with larger cohorts and comparable SSTR tracers must validate our results.

## Conclusion

This study demonstrated that ^99m^Tc-HYNIC-TOC SPECT/CT is an accurate imaging modality in the localization of culprit tumors responsible for TIO. This can provide clinicians with more comprehensive information for clinical diagnosis, treatment, and prognostic evaluation.

## Data availability statement

The original contributions presented in the study are included in the article/supplementary material. Further inquiries can be directed to the corresponding authors.

## Ethics statement

The studies involving human participants were reviewed and approved by the ethics committee of Henan Provincial People’s Hospital & Zhengzhou University People’s Hospital. The patients/participants provided their written informed consent to participate in this study.

## Author contributions

YG, XW, and BL designed the present study. BL, XW, LD, XC and YG collected and analyzed the data. JS, XL, HQL and HML analyzed the data. BL, YG and XW drafted the manuscript. YG and BL can authenticate all raw data. All the authors read and approved the final manuscript.

## References

[1] Minisola S Peacock M Fukumoto S Cipriani C Pepe J Tella SH. Tumour-induced osteomalacia. Nat Rev Dis Primers (2017) 3:17044. doi: 10.1038/nrdp.2017.44 28703220

[2] Jan de Beur SM Streeten EA Civelek AC McCarthy EF Uribe L Marx SJ. Localisation of mesenchymal tumours by somatostatin receptor imaging. Lancet (2002) 359:761–3. doi: 10.1016/s0140-6736(02)07846-7 11888589

[3] El-Maouche D Sadowski SM Papadakis GZ Guthrie L Cottle-Delisle C Merkel R. (68)Ga-DOTATATE for tumor localization in tumor-induced osteomalacia. J Clin Endocrinol Metab (2016) 101:3575–81. doi: 10.1210/jc.2016-2052 PMC5052344 27533306

[4] Bhavani N Reena Asirvatham A Kallur K Menon AS Pavithran PV Nair V. Utility of Gallium-68 DOTANOC PET/CT in the localization of Tumour-induced osteomalacia. Clin Endocrinol (Oxf) (2016) 84:134–40. doi: 10.1111/cen.12822 25996566

[5] Paquet M Gauthé M Zhang Yin J Nataf V Bélissant O Orcel P. Diagnostic performance and impact on patient management of (68)Ga-DOTA-TOC PET/CT for detecting osteomalacia-associated tumours. Eur J Nucl Med Mol Imaging (2018) 45:1710–20. doi: 10.1007/s00259-018-3971-x 29532101

[6] Clifton-Bligh RJ Hofman MS Duncan E Sim Ie W Darnell D Clarkson A. Improving diagnosis of tumor-induced osteomalacia with Gallium-68 DOTATATE PET/CT. J Clin Endocrinol Metab (2013) 98:687–94. doi: 10.1210/jc.2012-3642 23295468

[7] Breer S Brunkhorst T Beil FT Peldschus K Heiland M Klutmann S. 68Ga DOTA-TATE PET/CT allows tumor localization in patients with tumor-induced osteomalacia but negative 111In-octreotide SPECT/CT. Bone (2014) 64:222–7. doi: 10.1016/j.bone.2014.04.016 24769333

[8] Zhang J Zhu Z Zhong D Dang Y Xing H Du Y. 68Ga DOTATATE PET/CT is an accurate imaging modality in the detection of culprit tumors causing osteomalacia. Clin Nucl Med (2015) 40:642–6. doi: 10.1097/rlu.0000000000000854 26053726

[9] Singh D Chopra A Ravina M Kongara S Bhatia E Kumar N. Oncogenic osteomalacia: role of Ga-68 DOTANOC PET/CT scan in identifying the culprit lesion and its management. Br J Radiol (2017) 90:20160811. doi: 10.1259/bjr.20160811 28181822 PMC5605070

[10] Kato A Nakamoto Y Ishimori T Hayakawa N Ueda M Temma T. Diagnostic performance of (68)Ga-DOTATOC PET/CT in tumor-induced osteomalacia. Ann Nucl Med (2021) 35:397–405. doi: 10.1007/s12149-021-01575-x 33582980

[11] Lee DY Lee SH Kim BJ Kim W Yoon PW Lee SJ. Usefulness of (68)Ga-DOTATOC PET/CT to localize the culprit tumor inducing osteomalacia. Sci Rep (2021) 11:1819. doi: 10.1038/s41598-021-81491-2 33469091 PMC7815743

[12] Jing H Li F Zhuang H Wang Z Tian J Xing X. Effective detection of the tumors causing osteomalacia using [Tc-99m]-HYNIC-octreotide (99mTc-HYNIC-TOC) whole body scan. Eur J Radiol (2013) 82:2028–34. doi: 10.1016/j.ejrad.2013.04.006 23721625

[13] Zuo QY Wang H Li W Niu XH Huang YH Chen J. Treatment and outcomes of tumor-induced osteomalacia associated with phosphaturic mesenchymal tumors: retrospective review of 12 patients. BMC Musculoskelet Disord (2017) 18:403. doi: 10.1186/s12891-017-1756-1 28934935 PMC5609032

[14] Li B Duan L Shi J Han Y Wei W Cheng X. Diagnostic performance of 99mTc-HYNIC-PSMA SPECT/CT for biochemically recurrent prostate cancer after radical prostatectomy. Front Oncol (2022) 12:1072437. doi: 10.3389/fonc.2022.1072437 36568205 PMC9768541

[15] Jadhav S Kasaliwal R Lele V Rangarajan V Chandra P Shah H. Functional imaging in primary tumour-induced osteomalacia: relative performance of FDG PET/CT vs somatostatin receptor-based functional scans: a series of nine patients. Clin Endocrinol (Oxf) (2014) 81:31–7. doi: 10.1111/cen.12426 24528172

[16] Chong WH Andreopoulou P Chen CC Reynolds J Guthrie L Kelly M. Tumor localization and biochemical response to cure in tumor-induced osteomalacia. J Bone Miner Res (2013) 28:1386–98. doi: 10.1002/jbmr.1881 PMC3900247 23362135

[17] Decristoforo C Melendez-Alafort L Sosabowski JK Mather SJ. 99mTc-HYNIC-[Tyr3]-octreotide for imaging somatostatin-receptor-positive tumors: preclinical evaluation and comparison with 111In-octreotide. J Nucl Med (2000) 41:1114–9. 10855644

[18] Zhang Y Zhang X Zhang W Huang Z Chen Y. Diagnostic value of 68Ga-DOTA-TATE PET/CT imaging for tumor- induced osteomalacia. Ann Palliat Med (2020) 9:3350–6. doi: 10.21037/apm-20-1466 32921125

[19] Jagtap VS Sarathi V Lila AR Malhotra G Sankhe SS Bandgar T. Tumor-induced osteomalacia: a single center experience. Endocr Pract (2011) 17:177–84. doi: 10.4158/ep10151.Or 20713341

[20] Agrawal K Bhadada S Mittal BR Shukla J Sood A Bhattacharya A. Comparison of 18F-FDG and 68Ga DOTATATE PET/CT in localization of tumor causing oncogenic osteomalacia. Clin Nucl Med (2015) 40:e6–e10. doi: 10.1097/rlu.0000000000000460 24999675

[21] Jain AS Shelley S Muthukrishnan I Kalal S Amalachandran J Chandran S. Diagnostic importance of contrast enhanced (18)F-fluorodeoxyglucose positron emission computed tomography in patients with tumor induced osteomalacia: Our experience. Indian J Nucl Med (2016) 31:14–9. doi: 10.4103/0972-3919.172344 PMC4746834 26917888

[22] Yu HN Liu L Chen QS He Q Li YS Wang Y. Comparison of (18) F-FDG PET/CT and (68) ga-DOTATATE PET/CT in the targeted imaging of culprit tumors causing osteomalacia. Orthop Surg (2021) 13:791–8. doi: 10.1111/os.12980 PMC8126929 33709632

[23] Satyaraddi A Cherian KE Shetty S Kapoor N Jebasingh FK Cherian VM. Musculoskeletal oncogenic osteomalacia-An experience from a single centre in South India. J Orthop (2017) 14:184–8. doi: 10.1016/j.jor.2016.12.010 PMC5222952 28123260

[24] Zhang S Wang L Wang T Xing HQ Huo L Li F. [Value of (68)Ga-DOTA-TATE positron emission tomography/computed tomography in the localization of culprit tumors causing osteomalacia with negative (99m)Tc-HYNIC-TOC single photo emission computed tomography]. Zhongguo Yi Xue Ke Xue Yuan Xue Bao (2018) 40:757–64. doi: 10.3881/j.issn.1000-503X.10693 30606385

[25] Ding J Hu G Wang L Li F Huo L. Increased activity due to fractures does not significantly affect the accuracy of 68Ga-DOTATATE PET/CT in the detection of culprit tumor in the evaluation of tumor-induced osteomalacia. Clin Nucl Med (2018) 43:880–6. doi: 10.1097/rlu.0000000000002290 30273206

[26] Long T Hou J Yang N Zhou M Li Y Li J. Utility of 18F-alF-NOTA-octreotide PET/CT in the localization of tumor-induced osteomalacia. J Clin Endocrinol Metab (2021) 106:e4202–9. doi: 10.1210/clinem/dgab258 33864458

[27] Hou G Zhang Y Liu Y Wang P Xia W Xing X. Head-to-head comparison of (68)Ga-DOTA-TATE and (68)Ga-DOTA-JR11 PET/CT in patients with tumor-induced osteomalacia: A prospective study. Front Oncol (2022) 12:811209. doi: 10.3389/fonc.2022.811209 35280786 PMC8913035

[28] Ho CL. Ga68-DOTA peptide PET/CT to detect occult mesenchymal tumor-inducing osteomalacia: A case series of three patients. Nucl Med Mol Imaging (2015) 49:231–6. doi: 10.1007/s13139-015-0328-2 PMC4532688 26279697

[29] He Q Zhang B Zhang L Chen Z Shi X Yi C. Diagnostic efficiency of (68)Ga-DOTANOC PET/CT in patients with suspected tumour-induced osteomalacia. Eur Radiol (2021) 31:2414–21. doi: 10.1007/s00330-020-07342-2 33021702

[30] Xia X Shao F Hu F Gai Y Lan X. Culprit tumor as an unexpected extraosseous MDP activity on bone scintigraphy in a patient with tumor-induced osteomalacia. Clin Nucl Med (2020) 45:492–4. doi: 10.1097/rlu.0000000000003042 32366790

[31] Wang P Zhang S Huo L Jing H Li F. Prognostic value of positive presurgical FDG PET/CT in the evaluation of tumor-induced osteomalacia. Clin Nucl Med (2021) 46:214–9. doi: 10.1097/rlu.0000000000003463 33351512

[32] Bosman A Palermo A Vanderhulst J De Beur SMJ Fukumoto S Minisola S. Tumor-induced osteomalacia: A systematic clinical review of 895 cases. Calcif Tissue Int (2022) 111:367–79. doi: 10.1007/s00223-022-01005-8 PMC9474374 35857061

